# Inflammation of the Mucous Surface of the Fauces

**Published:** 1864-05

**Authors:** G. D. Winch

**Affiliations:** Asst. Surg. 36th Reg. Wis. Vols.


### INFLAMMATION OF THE MUCOUS SURFACE OF THE FAUCES.

By G. D. WINCH, Asst. Surg. 36th Reg. Wis. Vols.

CAMP RANDALL, MADISON, WIS.

For the last four weeks we have been overrun with sore throat. It occurs in those who have been exposed, by being on guard or standing on inspection without overcoats. The symptoms are, a feeling of dryness in the throat, little tenderness by external pressure, difficulty of swallowing, constant coughing, no acceleration of the pulse except in severe cases, no loss of appetite, but the pain in the parts is continued and severe.

Now in treatment of these cases, I have watched carefully, the effects of remedies. Sulph. Zinc, acetas plumbi, chlorate potassa, and capsicum have no effect in curing the disease. Such cases will recover as soon without treatment as with the above remedies. There is an irritability that keeps up the inflammation that they do not blunt. But let the saturated solution of argenti nitratis (forty grains to the ounce,) be applied, and after two applications the irritability is reduced, inflammation subsides, and the parts soon become normal.

With the above treatment, use measures to not have the patient's throat exposed to the cold air.

